# Pathological T3a Upstaging of Clinical T1 Renal Cell Carcinoma: Outcomes According to Surgical Technique and Predictors of Upstaging

**DOI:** 10.1371/journal.pone.0166183

**Published:** 2016-11-18

**Authors:** Seung-hwan Jeong, Jung Kwon Kim, Juhyun Park, Ho Joon Jeon, Min Young Yoon, Chang Wook Jeong, Ja Hyeon Ku, Hyeon Hoe Kim, Cheol Kwak

**Affiliations:** 1 Department of Urology, Seoul National University Hospital, Seoul, Korea; 2 Department of Urology, Seoul Metropolitan Government—Seoul National University Boramae Medical Center, Seoul National University College of Medicine, Seoul, Korea; National Health Research Institutes, TAIWAN

## Abstract

**Purpose:**

To evaluate the prognosis of pT3a upstaging from cT1 renal cell carcinoma, and to compare the outcomes of partial or radical nephrectomy in cases of pT3a upstaging.

**Materials and Methods:**

We reviewed the records of patients who underwent partial or radical nephrectomy for cT1 at our center between January 2001 and October 2013. We compared the 2-year recurrence-free survivals for cases with pT1 or pT3a upstaging, and for partial or radical nephrectomy in cases with pT3a upstaging. Clinicopathological parameters were analyzed in univariate and multivariate analyses to evaluate their associations with upstaging.

**Results:**

Among the 1,009 eligible patients, 987 patients were included in the analysis. The mean follow-up was 48.5 ± 27.8 months in whole patients. The 2-year recurrence-free survival was worse in the pT3a upstaging group, compared to the pT1 group (87.3% vs. 98.7%; p < 0.001). Partial nephrectomy and radical nephrectomy had no significant difference in 2-year recurrence-free survivals (91.9% vs. 83.7%; p = 0.251). The multivariate analysis revealed that upstaging was associated with old age, cT1b stage, clinical symptoms, and a high Fuhrman grade.

**Conclusions:**

Pathological T3a upstaging of cT1 renal cell carcinoma was associated with a poorer prognosis, compared to pT1 disease. However, the surgical technique (radical or partial nephrectomy) did not affect the recurrence rate. Therefore, clinicians should select the treatment method based on the clinical stage, and consider the pathological stage during the follow-up.

## Introduction

Partial nephrectomy is recommended for the treatment of T1 renal cell carcinoma (RCC), as it preserves renal function and provides oncological outcomes that are comparable to those of radical nephrectomy [1–3]. In cases of surgically treatable T2 RCC, partial nephrectomy can be performed, although it is not generally used for T3 RCC [4, 5]. Thus, clinical T stage is considered important for selecting the surgical technique (partial vs. radical nephrectomy), and is typically determined using computed tomography (CT). The American Joint Committed on Cancer (AJCC) established seventh TNM staging system which is based on the tumor size or depth of invasion (T), lymph node status (N) and metastasis (M). In this context, T1 and T2 tumors are limited to the kidney and are classified according to the tumor's size (≤7 cm or >7 cm, respectively). In contrast, T3a disease is defined as exhibiting perirenal fat invasion, renal sinus fat infiltration, or renal vein thrombosis, regardless of the tumor's size [6]. Fuhrman nuclear grading system is most widely used for estimating nuclear grade according to the three features of nuclear size, shape and nucleoli. The high grade tumors are associated with poor prognosis. However the Fuhrman grade is not included in determining treatment plans [7, 8].

The microscopic perirenal invasion, renal sinus fat infiltration, and renal vein thrombosis can be missed during CT, and pT3a upstaging occasionally occurs in cases of cT1 RCC [9–11]. Furthermore, previous studies have revealed conflicting findings regarding the prognoses and risk factors for T3a upstaging [12–15].

Therefore, the present study aimed to define the effect of pT3a upstaging from cT1 on recurrence-free survival, to evaluate the outcomes of pT3a upstaging according to surgical technique (partial or radical nephrectomy), and to identify the clinical factors that were associated with upstaging.

## Materials and Methods

This study’s retrospective design was approved by the institutional review board of the Seoul National University Hospital (Approval number: H-1604-039-753). We included consecutive patients who underwent partial nephrectomy for clinical T1N0M0 disease and radical nephrectomy exhibited pT3a up staging from clinical T1N0M0 disease between January 2001 and October 2013 at our institution. All surgical techniques were included (e.g., open, laparoscopic, and robotic surgeries). The patient records were anonymized and de-identified prior to analysis.

However, we excluded cases with non-RCC pathology, bilateral or multiple renal tumors, lymph node metastasis, or von Hippel-Lindau disease. Clinical T stage was assessed using contrast-enhanced CT, according to the seventh AJCC TNM staging system.

Patients were classified into three groups: pT3a upstaging after partial nephrectomy (group A, n = 37), pT3a upstaging after radical nephrectomy (group B, n = 54), and no pT3a upstaging after partial nephrectomy (group C, n = 896).

The clinicopathological characteristics that we evaluated included age, sex, body mass index (BMI), cT stage, clinical symptoms, tumor histology, Fuhrman grade, positive surgical margins, and pseudosarcomatous components. Clinical symptoms were defined as what patients suffered from or complained about such as hematuria, flank pain and a palpable mass. Tumor histology, Fuhrman grade, positive surgical margin and pseudosarcomatous components were estimated by pathologists. Fuhrman grade was classified from 1 to 4 according to uniformity of nuclear size, nuclear shape and nucleolar prominence. Pseudosarcomatous components were reported for tumors with sarcomatoid differentiation characterized by spindle cell histology [16].

Postoperative follow-up was performed using contrast-enhanced kidney CT and chest radiography at 6 months, and then annually thereafter.

The 2-year recurrence-free survivals in all groups were analyzed using the Kaplan-Meier method and the log rank test. Clinicopathological characteristics were compared using the Mann-Whitney U test for continuous variables and the chi-square test for categorical variables. Multivariate analyses were performed using logistic regression. All analyses were performed using SPSS software (version 19.0; SPSS Inc., Chicago, IL, USA), and differences were considered statistically significant at a two-sided p-value of <0.05.

## Results

A total of 987 patients were analyzed, and their clinicopathological characteristics are shown in Table 1. The mean follow-ups were 50.8 ± 32.4 months for groups A and B (upstaging) and 48.2 ± 27.2 months for group C (no upstaging). The upstaging groups were significantly older, compared to the no upstaging group (58.6 ± 13.9 years vs. 54.9 ± 12.6 years; p = 0.006). The upstaging groups also exhibited a higher proportion of cT1b stage, compared to the no upstaging group (47.3% vs. 10.9%; p < 0.001). Clinical symptoms (e.g., hematuria, flank pain, and a palpable mass) were significantly more common in the upstaging groups, compared to the no upstaging group (21.8% vs. 10.8%; p = 0.002). High-grade tumors (Fuhrman grade 3–4) were significantly more frequent in the upstaging groups (63.7% vs. 28.2%; p < 0.001). The upstaging groups exhibited a higher frequency of pseudosarcomatous components, compared to the no upstaging group (4.4% vs. 0.2%; p = 0.001). There were no significant differences in the tumor histology distributions or rates of positive surgical margins between the two groups.

**Table 1:** Clinical and pathological parameters.

Variable	No upstaging (N = 896)	Upstaging (N = 91)	P-value
Age (years), mean ± SD	54.9 ± 12.6	58.6 ± 13.9	0.006
Sex, no. (%)			0.712
Men	647 (72.2)	68 (74.7)	
Women	249 (27.8)	23 (25.3)	
BMI (kg/m^2^), mean ± SD	24.6 ± 3.2	24.7 ± 3.1	0.651
Clinical stage			< 0.001
T1a	798 (89.1)	48 (52.7)	
T1b	98 (10.9)	43 (47.3)	
Symptoms (%)	97 (10.8)	21 (21.8)	0.002
Follow-up (months), mean ± SD	48.2 ± 27.2	50.8 ± 32.4	0.752
Pathological T stage (%)		N.A.	
T1a	499 (89.2)		
T1b	97 (10.8)		
Histology (%)			0.134
Clear cell	721 (80.5)	69 (75.8)	
Papillary	81 (9.0)	7 (7.7)	
Chromophobe	69 (7.7)	11 (12.1)	
Other	25 (2.8)	4 (4.4)	
Fuhrman grade (%)			< 0.001
1	88 (9.8)	1 (1.1)	
2	553 (61.7)	32 (35.2)	
3	242 (27.0)	50 (54.9)	
4	11 (1.2)	8 (8.8)	
Positive surgical margin, no. (%)	33 (3.7)	2 (2.2)	0.764
Pseudosarcomatous component, no. (%)	2 (0.2)	4 (4.4)	0.001

SD: standard deviation; BMI: body mass index.

The results of the multivariate analyses are shown in Table 2. Upstaging was associated with old age (odds ratio [OR]: 1.026, 95% confidence interval [95% CI]: 1.007–1.046, p = 0.009), cT1b stage (OR: 5.882, 95% CI: 3.585–9.651, p < 0.001), clinical symptoms (OR: 2.330, 95% CI: 1.282–4.234, p = 0.006) and a high Fuhrman grade (grade 2, OR: 4.008, 95% CI: 0.535–30.027, p = 0.177; grade 3, OR: 12.206, 95% CI: 1.640–90.875, p = 0.015; grade 4, OR: 33.911, 95% CI: 3.520–327.647, p = 0.002). The presence of pseudosarcomatous components was not significantly associated with upstaging.

**Table 2:** Multivariate analyses of clinicopathological parameters that were associated with pT3a upstaging.

Variable	Odds ratio	95% CI	P-value
Age	1.026	1.007–1.046	0.009
Clinical stage			
T1a	1		
T1b	5.882	3.585–9.651	< 0.001
Symptoms	2.330	1.282–4.234	0.006
Fuhrman grade (%)			
1	1		
2	4.008	0.535–30.027	0.177
3	12.206	1.640–90.875	0.015
4	33.911	3.520–327.647	0.002
Pseudosarcomatous component	2.679	0.373–19.230	0.327

CI: confidence interval

**Fig 1:**
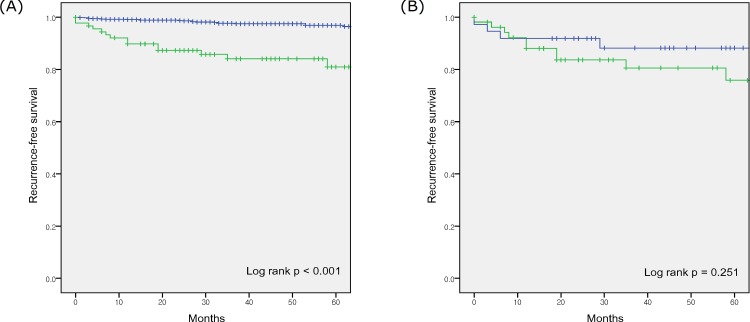
Kaplan-Meier survival curves for recurrence-free survivals. (A) the pT1 (blue curve) and pT3a upstaging groups (green curve), and (B) the partial nephrectomy (blue curve) and radical nephrectomy (green curve) subgroups of the pT3a upstaging group.

**Fig 2:**
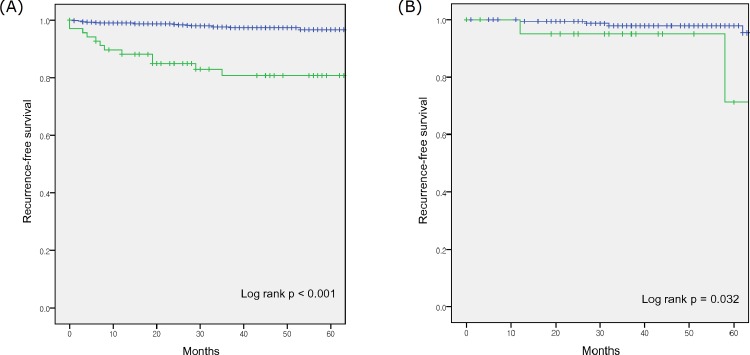
Kaplan-Meier survival curves for recurrence-free survivals. (A) the pT1 (blue curve) and pT3a upstaging groups (green curve) of the clear cell subtype, and (B) the pT1 (blue curve) and pT3a upstaging groups (green curve) of the non clear cell subtypes.

The no upstaging group exhibited a higher estimated 2-year recurrence-free survival, compared to the upstaging groups (98.7% vs. 87.3%; p < 0.001) (Fig 1). In the upstaging groups, 14 patients experienced distant metastasis and 1 patient experienced local recurrence. In the no upstaging group, 19 patients experienced distant metastasis and 6 patients experienced local recurrence. In the subgroup analysis according to histological subtype, the no upstaging group with clear cell histology experienced a higher estimated 2-year recurrence-free survival, compared to the upstaging group (98.6% vs. 84.9%; p < 0.001) (Fig 2). Among the non-clear cell histological subtypes, the no upstaging group experienced a higher estimated 2-year recurrence-free survival, compared to the upstaging group (99.4% vs. 95.0%; p = 0.032).

The estimated 2-year recurrence-free survivals were not statistically different between groups A and B (91.9% vs. 83.7%, respectively; p = 0.251) (Fig 1). Group B had a significantly higher proportion of cT1b stage, compared to group A (63.0% vs. 24.3%; p = 0.001), although the rates of clinical symptoms were not significantly different between groups A and B (16.2% vs. 27.8%; p = 0.218) (S1 Table).

## Discussion

Partial nephrectomy has been the standard treatment for T1 RCC, because it provides extended overall survival that is related to preserved renal function and reduced cardiovascular risk [2, 3, 5]. Furthermore, given that it provides oncological outcomes that are equivalent to those of radical nephrectomy, attempts have been made to use partial nephrectomy for T2 RCC [5, 17]. However, in locally advanced RCC, violation of the Gerota's fascia and dissection of the perirenal fat during partial nephrectomy can increase the risk of recurrence [5].

The TNM stage typically determines the treatment option, follow-up protocol, and prognosis [18, 19]. Clinical staging is typically performed using contrast-enhanced CT, although there is a risk of missing renal sinus fat invasion, perirenal fat invasion, or renal vein thrombosis during CT, which can lead to pT3a upstaging [9, 11, 20]. Sokhi et al. reported the sensitivity of CT for sinus fat, perirenal fat and renal vein invasion up to 88, 83, 69% respectively [9]. Previously conducted studies reported T3a upstaging rate of 13.3–30.7% [12–14].

Upstaging occurred in 9.2% of our patients although it was lower than the rates of previous studies. The sinus fat invasion, perirenal fat invasion and renal vein thrombosis were observed in 23, 72 and 4 cases respectively.

The estimated 2-year recurrence-free survival rate was lower for pT3a upstaging, compared to pT1 disease (87.3% vs. 98.7%; p < 0.001). Gorin et al. reported similar findings, with a lower 24-month recurrence-free survival rate after robotic partial nephrectomy for cT1 in cases with pT3a upstaging, compared to cases with pT1 or pT2 disease (91.8% vs. 99.2%, p = 0.003) [12].

In contrast, Roberts et al. found that there was no significant difference in the 5-year recurrence-free survival rates in cases of pT3a upstaging or pT1 disease (90.6% vs. 97.5%, p = 0.08) [14]. Furthermore, Ramaswamy et al. reported that the oncological outcomes of pT3a upstaging from cT1 were good, because they did not observe recurrence in 66 patients with pT3a upstaging during a median follow-up of 50 months [13].

In the present study, the estimated 2-year recurrence-free survivals were not significantly different between groups A and B (91.9% vs. 83.7%; p = 0.251). Weight et al. analyzed the cancer-specific survivals among patients who underwent radical or partial nephrectomy with cT1 and pT3 upstaging, and also found equivalent survivals in the radical and partial nephrectomy groups [21]. Moreover, Hansen et al. found that partial and radical nephrectomy provided similar cancer-specific survivals among patients with pT3a disease. The 2- and 5- year cancer specific mortalities were 2.1 and 5.1% for partial nephrectomy and 3.0 and 6.0% for radical nephrectomy (p = 0.4) [22].

In the cases with pT3a upstaging, we observed distant metastasis in 14 patients (93.3%) and local recurrence in 1 patient (6.7%). In the pT1 group, we observed distant metastasis in 19 patients (76%) and local recurrence in 6 patients (24%). The rates of positive surgical margins were 2.2% in the pT3a group and 3.7% in the pT1 group. These findings suggest that tumors with pT3a upstaging tend to progress as distant metastasis, rather than local recurrence, and that progression is not typically related to failed local control. Moreover, the greater rates for high Fuhrman grades in the upstaging groups reflects the aggressive tumor biology of upstaged tumors, and accounts for the higher recurrence rate, compared to cases of pT1 disease.

It has been demonstrated that the clear cell subtype exhibits lower recurrence-free survival or cancer specific survival rates in localized RCC, compared to the papillary or chromophobe subtypes [23, 24]. In the present study, the trend towards poorer recurrence free survival was more pronounced in the clear cell subtype, compared to the non clear cell subtypes. These findings suggest that the clear cell subtype progresses more aggressively when it is associated with upstaging, compared to the non clear cell subtypes.

In the multivariate analyses, pT3a upstaging was associated with old age, cT1b stage, clinical symptoms, and a high Fuhrman grade. In addition, Ramaswamy et al. demonstrated that upstaging was associated with clear cell histology, a tumor size of >4 cm, and positive surgical margins [13]. Moreover, Tay et al. found that high RENAL nephrometry scores were a risk factor for upstaging, although age and Fuhrman grade were not significant risk factors [25].

The important implications of our findings are that tumors with pT3a upstaging have aggressive features (vs. pT1 tumors), and that the surgical technique (radical or partial nephrectomy) does not alter the prognosis. Therefore, clinicians do not have to avoid partial nephrectomy based on concerns regarding upstaging, although cautious follow-up is warranted in cases with upstaging. Furthermore, the risk of upstaging should be considered preoperatively in cases that involve old age, cT1b stage, or clinical symptoms.

There are several limitations in the present study. First, this study used a single-center retrospective design, which is associated with a well-known risk of biases such as selection bias and information bias. Second, this study had a small sample size. Third, we only evaluated the 2-year recurrence-free survivals, and did not analyze long-term outcomes. However this study is the first study showing the effects of upstaging and surgical technique together and comparing the prognoses of upstaging according to histological subtypes.

The large prospective multicenter randomized cohort studies are needed to validate our findings.

## Conclusions

The postoperative recurrence-free survival in cases of cT1 RCC was worse in cases with pT3a upstaging, compared to cases with pT1 disease. Furthermore, old age, cT1b stage, clinical symptoms, and a high Fuhrman grade were associated with pT3a upstaging. Therefore, because partial nephrectomy and radical nephrectomy have no significant difference in recurrence-free survivals, the treatment plan should be determined based on clinical stage and operability.

## Supporting Information

S1 Table Clinical and pathological parameters of upstaging tumors according to surgical technique. (DOCX)
