# Supplementary material for: Pathological T3a Upstaging of Clinical T1 Renal Cell Carcinoma: Outcomes According to Surgical Technique and Predictors of Upstaging
Source: PLoS One. 2016 Nov 18;11(11):e0166183. doi: 10.1371/journal.pone.0166183 (PMC5115709; doi:10.1371/journal.pone.0166183)
Supplement: S1 Table — (DOCX) [file pone.0166183.s001.docx]

S1 Table. Clinical and pathological parameters of upstaging tumors according to surgical technique.

| Variable | Group A  N = 37 | Group B  N = 54 | P-value |
| --- | --- | --- | --- |
| Age (years), mean ± SD | 54.9 ± 15.0 | 61.1 ± 12.7 | 0.041 |
| Sex, no. (%)  Men  Women | 26 (70.3)  11 (29.7) | 42 (77.8)  12 (22.2) | 0.467 |
| BMI (kg/m^2^), mean ± SD | 25.0 ± 2.9 | 24.5 ± 3.2 | 0.529 |
| Clinical stage  T1a  T1b | 28 (75.7)  9 (24.3) | 20 (37.0)  34 (63.0) | 0.001 |
| Symptoms (%) | 6 (16.2) | 15 (27.8) | 0.218 |
| Follow-up (months), mean ± SD | 48.5 ± 24.2 | 52.4 ± 37.2 | 0.992 |
| Histology (%)  Clear cell  Papillary  Chromophobe  Other | 28 (75.7)  3 (8.1)  5 (13.5)  1 (2.7) | 41 (75.9)  4 (7.4)  6 (11.1)  3 (5.6) | 0.947 |
| Fuhrman grade (%)  1  2  3  4 | 1 (2.7)  15 (40.5)  20 (54.1)  1 (2.7) | 0  17 (31.5)  30 (55.6)  7 (13.0) | 0.169 |
| Positive surgical margin, no. (%) | 2 (5.4) | 0 | 0.163 |
| Pseudosarcomatous component, no. (%) | 1 (2.7) | 3 (5.6) | 0.643 |

SD: standard deviation; BMI: body mass index.
